# Associação entre o Antagonismo do Sistema Renina-Angiotensina-Aldosterona e a Mortalidade Relacionada à COVID-19 em Pacientes com Hipertensão Relacionada ao Sobrepeso/Obesidade: um Estudo Retrospectivo de Coorte

**DOI:** 10.36660/abc.20220277

**Published:** 2023-03-24

**Authors:** Elham Shams, Vijayvardhan Kamalumpundi, Linhai Cheng, Adeyinka Taiwo, Amal Shibli-Rahhal, Ayotunde O. Dokun, Marcelo L.G. Correia

**Affiliations:** 1 Division of Endocrinology and Metabolism Department of Internal Medicine University of lowa EUA Division of Endocrinology and Metabolism, Department of Internal Medicine, University of lowa – EUA; 2 Carver College of Medicine University of Iowa EUA Carver College of Medicine, University of Iowa - EUA

**Keywords:** Antagonistas de Receptores de Angiotensina, Inibidores da Enzima Conversora de Angiotensina, COVID-19, Obesidade, Hipertensão

## Abstract

**Fundamento:**

Os bloqueadores dos receptores da angiotensina (BRA) e os inibidores da enzima conversora da angiotensina (IECA) aumentam a expressão de ACE2, que é um receptor para entrada de SARS-CoV-2 nas células. Embora as evidências sugiram que os IECA/BRA são seguros entre a população geral com COVID-19, sua segurança em pacientes com hipertensão relacionada ao sobrepeso/obesidade merece uma avaliação mais aprofundada.

**Objetivo:**

Avaliamos a associação entre o uso de IECA/BRA e a gravidade da COVID-19 em pacientes com hipertensão relacionada ao sobrepeso/obesidade.

**Métodos:**

O presente estudo incluiu 439 pacientes adultos com sobrepeso/obesidade (índice de massa corporal ≥ 25 kg/m^2^) e hipertensão, diagnosticados com COVID-19 e internados no University of Iowa Hospitals and Clinic entre 1º de março e 7 de dezembro de 2020. Foram avaliadas a mortalidade e a gravidade da COVID-19 com base no tempo de internação hospitalar, internação em unidade de terapia intensiva, uso de oxigênio suplementar, ventilação mecânica e uso de vasopressores. A regressão logística multivariável foi usada para examinar as associações do uso de IECA/BRA com a mortalidade e outros marcadores de gravidade de COVID-19, com um alfa bilateral definido em 0,05.

**Resultados:**

A exposição aos BRA (n = 91) e IECA (n = 149) antes da hospitalização foi significativamente associada a menor mortalidade ( *odds ratio* [OR] = 0,362, intervalo de confiança [IC] de 95% 0,149 a 0,880, p = 0,025) e menor tempo de internação hospitalar (IC 95% −0,217 a −0,025, p = 0,015). Adicionalmente, os pacientes em uso de IECA/BRA apresentaram uma tendência não significativa de menor internação em unidade de terapia intensiva (OR = 0,727, IC 95% 0,485 a 1,090, p = 0,123), uso de oxigênio suplementar (OR = 0,929, IC 95% 0,608 a 1,421,p = 0,734), ventilação mecânica (OR = 0,728, IC 95% 0,457 a 1,161, p = 0,182) e vasopressores (OR = 0,677, IC 95% 0,430 a 1,067, p = 0,093).

**Conclusão:**

Os resultados sugerem que pacientes internados com COVID-19 e hipertensão relacionada ao sobrepeso/obesidade que receberam IECA/BRA antes da internação apresentam menor mortalidade e COVID-19 menos grave do que aqueles que não estavam tomando IECA/BRA. Os resultados também sugerem que a exposição aos IECA/BRA pode proteger pacientes com hipertensão relacionada ao sobrepeso/obesidade de COVID-19 grave e morte.

**Figura Central:**
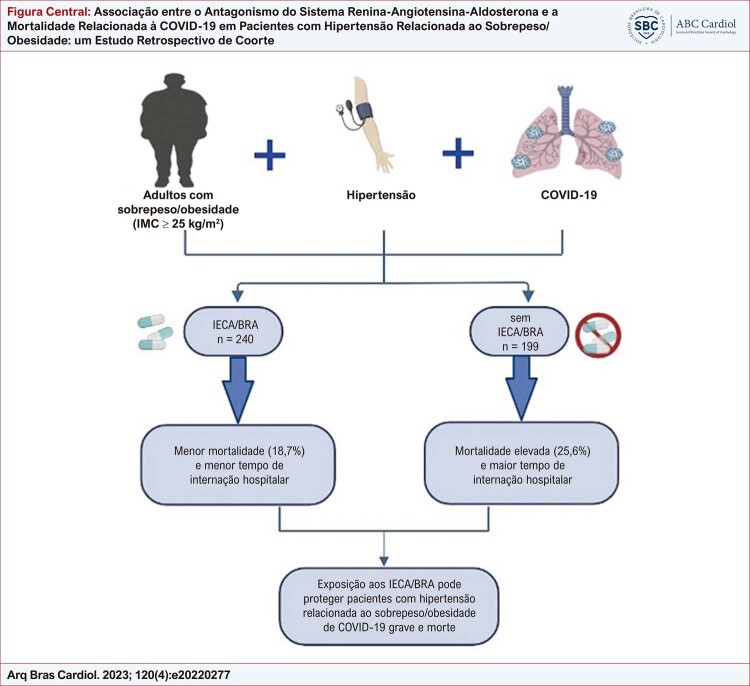
: Associação entre o Antagonismo do Sistema Renina-Angiotensina-Aldosterona e a Mortalidade Relacionada à COVID-19 em Pacientes com Hipertensão Relacionada ao Sobrepeso/Obesidade: um Estudo Retrospectivo de Coorte

IECA/BRA: inibidores da enzima conversora da angiotensina/bloqueadores dos receptores da angiotensina; IMC: índice de massa corporal.

## Introdução

A pandemia da doença de coronavírus 2019 (COVID-19) foi causada por um novo vírus de RNA de fita simples de sentido positivo chamado coronavírus da síndrome respiratória aguda grave 2 (SARS-CoV-2). Até o momento, a COVID-19 já foi responsável pela morte de mais de 6,3 milhões de vidas em todo o mundo, com os Estados Unidos liderando o mundo em número absoluto de mortes.^1 - 3^ Um crescente corpo de evidências sugere que a idade avançada (≥ 65 anos) e comorbidades como hipertensão, diabetes e obesidade são fatores de risco independentes para COVID-19 mais grave em comparação com a população geral.^2 , 4^

O mecanismo de entrada viral pelo SARS-CoV-2 tem sido bem caracterizado.^2 , 3 , 5^ O novo vírus explora a aminopeptidase ligada à membrana celular, enzima conversora da angiotensina 2 (ACE2) ligada à membrana celular para entrada e replicação viral.^2^ Evidências mecanísticas em modelos experimentais de camundongos selecionados mostraram que a expressão de ACE2 é regulada positivamente com a administração dos inibidores da enzima conversora da angiotensina /bloqueadores dos receptores da angiotensina (IECA/BRA).^5^ Esse mecanismo inicialmente levou muitos clínicos e pesquisadores a postularem que os IECA/BRA, ambos os quais bloqueiam o sistema renina-angiotensina-aldosterona (SRAA), podem aumentar a gravidade da infecção por COVID-19, promovendo a ligação do SARS-CoV-2 e a entrada celular. De fato, os dados clínicos atuais sugerem de forma esmagadora que os IECA/BRA não aumentam a gravidade e a mortalidade da COVID-19; porém, até o momento, nenhum estudo foi realizado exclusivamente em uma população com sobrepeso/obesidade.

É bem conhecido que pacientes com obesidade têm atividade aumentada do SRAA, com adipócitos no tecido adiposo visceral altamente expressando ECA2.^6^ Além disso, dada a desregulação metabólica, estado pró-inflamatório e taxas mais altas de trombose, a obesidade é conhecida por aumentar o risco de COVID-19 grave e mortalidade relacionada à COVID-19. Em um estudo recente entre 6.760 profissionais de saúde hospitalizados com COVID-19 em 14 estados dos Estados Unidos, a obesidade (72,5%) foi a comorbidade mais prevalente.^4^ Da mesma forma, em um estudo de coorte retrospectivo de centro único realizado na França com 124 pacientes com COVID-19 internados em terapia intensiva, o risco de ventilação mecânica invasiva foi quase 7 vezes maior para aqueles com índice de massa corporal (IMC) > 35 kg/m^2^ .^7^ Além da obesidade, hipertensão e outras comorbidades cardiovasculares são as comorbidades mais comuns em pacientes com COVID-19.^8^ Vários estudos observacionais multicêntricos e estudos populacionais demonstraram que pacientes com hipertensão apresentam COVID-19 mais grave e maior mortalidade.^8 - 10^

Vários estudos têm mostrado que IMC mais alto está relacionado a uma maior prevalência de distúrbios cardiovasculares, como hipertensão, acidente vascular cerebral, infarto do miocárdio e doença isquêmica do coração. Além disso, a maioria dos pacientes hospitalizados com COVID-19 grave sofria de comorbidades relacionadas ao excesso de adiposidade, como diabetes e distúrbios cardiovasculares,^11^ o que também está de acordo com relatos anteriores de surtos de influenza H1N1.^12^ Em um estudo retrospectivo com 1.965 pacientes com diabetes, em que 726 (36,9%) pacientes apresentavam sobrepeso e 805 (41,0%) obesidade, os autores relataram que essas condições foram associadas a mau prognóstico em pacientes com diabetes tipo 2 hospitalizados por COVID-19.^13^

O SRAA consiste em uma cascata enzimática responsável pelo controle da pressão arterial, mantendo o equilíbrio hidroeletrolítico e preservando a resistência vascular sistêmica.^3^ A cascata se inicia quando o angiotensinogênio plasmático é clivado pela renina, em angiotensina I (ang I). A enzima conversora de angiotensina então catalisa ang I em angiotensina II (ang II).^1^ A ECA2 usa ang II como substrato e produz ang (1-7). Ang (1-7) é um hormônio metabolicamente ativo que atua no receptor MAS para baixar a pressão arterial e possui propriedades anti-inflamatórias e antifibróticas. Os IECA inibem a formação de ang II a partir de ang I. Esse evento leva à conversão de ang I em um hormônio semelhante, ang 1-9. Ang 1-9 é rapidamente convertido em ang 1-7 por ECA2. Os BRA impedem que o ang II se ligue ao seu receptor; portanto, os ARBs inibem o efeito do ang II.^2 , 3^

Os IECA/BRA são usados extensivamente em pacientes com hipertensão, outras doenças cardiovasculares e diabetes para tratar pressão alta, insuficiência cardíaca, doença renal crônica e muitas outras doenças.^14^ Essas doenças geralmente são comorbidades com a obesidade; portanto, dados clínicos sobre o uso desses medicamentos em pacientes com hipertensão relacionada ao sobrepeso/obesidade são necessários. Para adicionar à base de evidências atuais acerca dos resultados clínicos desse grupo demográfico, foi realizado um estudo de coorte retrospectivo para investigar se existe uma associação entre a exposição aos IECA/BRA antes da internação e a gravidade da COVID-19 em pacientes com sobrepeso/obesidade relacionado à hipertensão.

## Métodos

### Fonte de dados e pacientes

Trata-se de um estudo de coorte retrospectivo de centro único realizado em pacientes diagnosticados com COVID-19 que foram internados no University of Iowa Hospitals and Clinic (UIHC). Foram usados os registros médicos eletrônicos da University of Iowa (Epic 2018, versão UI 2, Epic Systems Corporation, Verona, WI, EUA) para identificar todos os pacientes que (1) tinham idade ≥ 18 anos, (2) tinham IMC ≥ 25 kg/m^2^ e (3) apresentavam diagnóstico de hipertensão, internados na UIHC entre 1º de março e 7 de dezembro de 2020 com COVID-19. Os registros médicos eletrônicos contêm dados demográficos, clínicos, laboratoriais e de medicamentos completos de todos os pacientes atendidos em nosso centro médico. Foram excluídos pacientes com registro médico eletrônico incompleto e diagnóstico de hipertensão gestacional, pulmonar, portal, renal ou secundária. Foram incluídos no estudo pacientes que pararam de tomar medicamentos para o tratamento da hipertensão por qualquer motivo (por exemplo, aumento do nível de creatinina, incapacidade de tomar medicamentos por via oral devido à ventilação mecânica, etc.) durante a internação hospitalar. Adicionalmente, foram excluídos os pacientes em uso de IECA/BRA para qualquer outra indicação além de hipertensão (por exemplo, microalbuminúria relacionada ao diabetes).

### Elementos de dados

Os elementos de dados incluíram: idade, IMC, sexo, histórico de tabagismo/uso de álcool, comorbidades (por exemplo, diabetes e apneia obstrutiva do sono [AOS]), complicações durante a internação hospitalar (por exemplo, pancreatite, acidente vascular cerebral, síndrome do desconforto respiratório agudo, hepatite, etc.), tratamentos para COVID-19 (por exemplo, remdesivir, dexametasona, azitromicina, cloroquina, hidroxicloroquina), suporte ventilatório (por exemplo, oxigênio suplementar, oxigenação por membrana extracorpórea, ventilação invasiva, ventilação não invasiva), admissão em unidade de terapia intensiva (UTI), uso de vasopressores, mortalidade hospitalar e tempo de internação. O diagnóstico de COVID-19 foi determinado por teste positivo usando *swabs* nasofaríngeos, com uso raro de swabs orofaríngeos e escarro. Todas as amostras foram coletadas em meio de transporte viral não ativador de vários fabricantes com swabs flocados de alta qualidade (Center for Disease Control 2019-nCoV Real-Time RT-PCR Diagnostic Panel, Center for Disease Control Emergency Operations Center, Atlanta, GA, EUA; TaqPATH COVID-19 Combo kit, Cat#: A47814, Thermo Fisher Scientific Inc., Waltham, MA, EUA). O uso de IECA/BRA foi definido como o uso desses medicamentos no momento da admissão que foi interrompido durante a internação. De acordo com a medicação anti-hipertensiva, os pacientes foram divididos em 2 subgrupos, os que receberam prescrição de IECA/BRA antes da admissão ao hospital e os que não receberam prescrição de IECA/BRA (ou seja, o grupo de comparação). Todos os dados foram extraídos dos registros médicos eletrônicos dos pacientes por 3 investigadores (ES, VK, LC) usando um formulário de coleta de dados padronizado que foi subsequentemente verificado por 4 investigadores (ES, VK, LC, MC).

### Análise estatística

A análise estatística foi realizada com o software SPSS (IBM SPSS Statistics for Windows, Versão 27.0. Armonk, NY, EUA). Foram usadas estatísticas descritivas para resumir as características demográficas e clínicas dos pacientes. O teste de Kolmogorov-Smirnov foi utilizado para avaliar a distribuição das variáveis. As variáveis categóricas foram apresentadas como números e porcentagens e comparadas usando o teste qui-quadrado. As variáveis contínuas foram apresentadas como média e desvio padrão. Testes t de amostras independentes foram usados para comparar variáveis contínuas. A análise de regressão logística binária foi usada para analisar desfechos dependentes binários, como mortalidade, internação em UTI, uso de oxigênio suplementar, ventilação mecânica e vasopressores. Todos os modelos incluíram o uso de IECA/BRA como variável explicativa e foram ajustados para um conjunto de covariáveis, determinadas *a priori* , que poderiam confundir a associação entre o uso de IECA/BRA e a gravidade da doença. Essas covariáveis independentes incluíram idade, sexo, IMC, histórico de tabagismo, AOS, diabetes e uso de remdesivir e dexametasona. Foram realizados testes para interação entre as variáveis independentes. Modelos de regressão linear multivariada foram usados para desfechos contínuos, como tempo de internação. Os resultados foram apresentados *como odds ratio* (ORs) com intervalo de confiança de 95% (IC 95%) e valor de p ajustado. Foram considerados estatisticamente significativos valores de p abaixo de um alfa bilateral de 0,05.

No momento em que o presente estudo foi realizado, a Sociedade de Doenças Infecciosas da América (IDSA, sigla em inglês) já havia recomendado remdesivir para pacientes no estágio inicial da COVID-19 com a finalidade de reduzir a replicação viral, e dexametasona para pacientes em estágios avançados da doença com a finalidade de reduzir a produção de citocinas pró-inflamatórias.^15^ Há fortes evidências de que remdesivir e dexametasona podem alterar o curso da COVID-19.^16 , 17^ Portanto, essas variáveis foram adicionadas ao modelo como covariáveis pós-tratamento para determinar se houve um efeito da exposição IECA/BRA independente dos efeitos de remdesivir e/ou dexametasona. Não foram coletados dados sobre outros antibióticos além da azitromicina. Adicionalmente, os dados sobre o uso de hidroxicloroquina/cloroquina e azitromicina mais hidroxicloroquina para reduzir a mortalidade relacionada à COVID-19 foram considerados inconsistentes e, portanto, não adicionados ao modelo, mas foram coletados e incluídos em estatísticas descritivas.

## Resultados

### Características basais

**Figura 1:**
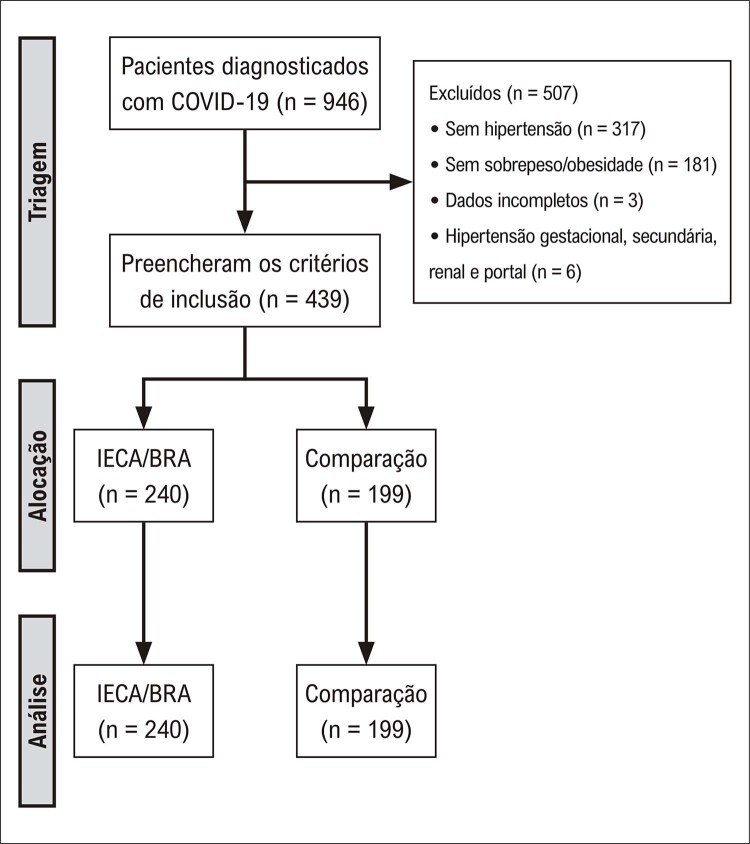
– Fluxograma do estudo. Critérios de inclusão: idade ≥ 18 anos, diagnóstico de COVID-19, hipertensão e IMC ≥ 25 kg/m2. BRA: bloqueador do receptor de angiotensina; COVID-19: doença de coronavírus 2019; IECA: inibidor da enzima conversora de angiotensina.

O presente estudo identificou 946 pacientes com COVID-19 confirmada que foram internados na UIHC. Após a aplicação dos critérios de exclusão, foram incluídos 439 pacientes na análise final ( Figura 1 ). Esta coorte foi subsequentemente agrupada no grupo IECA/BRA (n = 240) e no grupo de comparação (n = 199). Os dados demográficos dos pacientes são mostrados na Tabela 1 , e seus resultados e complicações são mostrados na Tabela 2 . As idades médias foram semelhantes em ambos os grupos. Em relação ao grupo de comparação, os pacientes que usavam IECA/BRA tinham uma proporção significativamente maior de homens e uma taxa mais alta de diabetes mellitus. A taxa de AOS em pacientes em uso de IECA/BRA foi maior do que no grupo de comparação, mas essa diferença não foi estatisticamente significativa (p = 0,186). Além disso, havia mais fumantes atuais no grupo de comparação do que no grupo IECA/BRA.

**Tabela 1:** – Dados demográficos de pacientes com hipertensão relacionada ao sobrepeso/obesidade entre os grupos IECA/BRA e comparação

	IECA/BRA (n = 240)	Comparação (n = 199)	Valor p
**Demográficos**			
Idade, anos (DP)	61,9 (13,3)	63,2 (15,1)	0,358
IMC (DP)	34,6 (8,4)	34,3 (7,9)	0,765
**Sexo**			
Masculino (%)	181 (75,4)	116 (58,2)	<0,001
**Comorbidades crônicas, n (%)**			
Diabetes	157 (65,4)	88 (44,2)	<0,001
AOS	74 (30,8)	50 (25,1)	0,186
Uso de CPAP	49 (20,4)	23 (11,5)	0,013
**Status tabágico, n (%)**			
Nunca fumante	100 (41,6)	87 (43,7)	
Ex-fumante	107 (44,5)	92 (46,2)	
Fumante atual	10 (4,1)	10 (5,0)	0,289^*^
**Tratamento, n (%)**			
Remdesivir	83 (34,5)	72 (36,1)	0,727
Plasma convalescente	33 (13,7)	31 (15,5)	0,589
Dexametasona	123 (51,2)	107 (53,7)	0,599
Cloroquina	0 (0)	0 (0)	--
Azitromicina	33 (13,7)	24 (12,0)	0,6
Hidroxicloroquina	2 (0,8)	1 (0,5)	1

Foram calculados os valores de p a partir de testes t de amostras independentes e testes qui-quadrado, quando apropriado. *O valor de p para fumantes foi calculado entre as categorias de fumante (atual e desconhecido) e não fumante (ex-fumante). AOS: apneia obstrutiva do sono; BRA: bloqueador dos receptores da angiotensina; CPAP: pressão positiva contínua nas vias aéreas; DP: desvio padrão; IECA: inibidor da enzima conversora da angiotensina; IMC: índice de massa corporal.

**Tabela 2:** – Desfechos e complicações de pacientes com hipertensão relacionada ao sobrepeso/obesidade entre os grupos IECA/BRA e comparação

	IECA/BRA (n = 240)	Comparação (n = 199)	Valor p
**Desfecho, n (%)**			
Oxigênio suplementar	158 (65,8)	130 (65,3)	0,911
Ventilação não invasiva	125 (52,0)	106 (53,2)	0,805
ECMO	3 (1,2)	6 (3,0)	0,311
Terapia intensiva	95 (39,5)	86 (43,2)	0,441
Ventilação mecânica	54 (22,5)	51 (25,6)	0,444
Vasopressores	57 (23,7)	56 (28,1)	0,295
Mortalidade	45 (18,7)	51 (25,6)	0,083
**Complicações, N (%)**			
Doença respiratória	78 (32,5)	54 (27,1)	0,222
Doenças renais e do trato urinário	34 (14,1)	21 (10,5)	0,255
Doença hepática	8 (3,3)	3 (1,5)	0,359
Doença cardiovascular	13 (5,4)	15 (7,5)	0,365
Doença pancreática	2 (0,8)	1 (0,5)	1
Doença hematológica	9 (3,7)	7 (3,5)	0,897
Choque séptico	10 (4,1)	6 (3,0)	0,522
Glicemia elevada	2 (0,8)	2 (1,0)	1
Encefalopatia aguda	8 (3,3)	7 (3,5)	0,916

Foram calculados os valores de p a partir de testes t de amostras independentes e testes qui-quadrado, quando apropriado. BRA: bloqueador dos receptores da angiotensina; ECMO: oxigenação por membrana extracorpórea; IECA: inibidor da enzima conversora da angiotensina.

### Medicamentos administrados no hospital

Entre os medicamentos administrados para tratamento de COVID-19, menos pacientes receberam dexametasona no grupo IECA/BRA em relação ao grupo de comparação, embora essa diferença não tenha sido estatisticamente significativa ( Tabela 1 ).

### Associações não ajustadas entre IECA/BRA e desfechos primários

A taxa de mortalidade não ajustada foi menor no grupo IECA/BRA do que no grupo de comparação (18,7% versus 25,6%, p = 0,083). Além disso, em relação ao grupo de comparação, os pacientes do grupo IECA/BRA apresentaram menor taxa de internação na UTI e menor uso de ventilação mecânica e vasopressores. Em geral, sem ajuste para outras covariáveis, a gravidade da COVID-19 para pacientes no grupo de comparação foi pior do que no grupo IECA/BRA.

### Associações ajustadas entre IECA/BRA e mortalidade

Após ajuste estatístico por idade, sexo, IMC, histórico de tabagismo, AOS, diabetes e uso de dexametasona e remdesivir, o grupo IECA/BRA apresentou chances significativamente menores de mortalidade do que os pacientes do grupo de comparação ( Tabela 3 ). Adicionalmente, os resultados dos modelos ajustados mostraram que a idade avançada e o sexo masculino foram associados ao aumento das chances de mortalidade relacionada à COVID-19. A interação entre o uso de IECA/BRA e diabetes não foi significativa. Além disso, não houve associação entre AOS e mortalidade, e não houve interação entre o uso de IECA/BRA e AOS na mortalidade.

**Tabela 3:** – Análise de regressão logística dos preditores de mortalidade entre pacientes com COVID-19 e hipertensão relacionada ao sobrepeso/obesidade nos grupos IECA/BRA e comparação

Variáveis	Odds ratio	Valor p
Idade	1,030 (1,010–1,051)	0,003
IMC	0,985 (0,950–1,022)	0,416
Sexo (masculino)	2,029 (1,149–3,584)	0,015
Uso de IECA/BRA	0,362 (0,149–0,880)	0,025
Diabetes	1,701 (0,860–3,364)	0,127
IECA ou BRA por diabetes	1,569 (0,532– 4,624)	0,414
AOS	1,237 (0,710–2,157)	0,453
Status tabágico: atual/não registrado	2,935 (1,520–5,665)	0,001
Dexametasona: sim	0,970 (0,567–1,659)	0,910
Remdesivir: sim	1,298 (0,755–2,233)	0,345

As variáveis preditoras foram codificadas da maneira seguinte: feminino = 0, masculino = 1; controle = 0, em uso IECA/BRA = 1; sem diabetes = 0, diabetes = 1; sem AOS = 0, AOS = 1; status tabágico: nunca/ex-fumante = 0, atual fumante/não registrado = 1; não tomou dexametasona = 0, tomou dexametasona = 1; não tomou remdesivir = 0, tomou remdesivir = 1; sobrevivência = 0, mortalidade = 1. Código 0 considerado como referência. Os números entre parênteses são intervalos de confiança de 95%. AOS: apneia obstrutiva do sono; BRA: bloqueador do receptor de angiotensina; IECA: inibidor da enzima conversora de angiotensina; IMC: índice de massa corporal.

### Associações ajustadas entre o uso de IECA/BRA e o tempo de internação hospitalar

Todos os parâmetros atenderam aos pressupostos necessários para a regressão linear. O modelo de regressão linear multivariada mostrou que os pacientes do grupo IECA/BRA tiveram menor tempo de internação após ajuste para idade, sexo, IMC, diabetes, AOS, status tabágico e uso de dexametasona e remdesivir ( Tabela 4 ).

**Tabela 4:** – Análise de regressão logística de preditores de tempo de internação entre pacientes com COVID-19 e hipertensão relacionada ao sobrepeso/obesidade nos grupos IECA/BRA e comparação

Variáveis	B padronizado	Valor p
Idade	0,002 (-0,096 a 0,100)	0,962
IMC	-0,056 (-0,160 a 0,048)	0,288
Sexo (masculino)	0,002 (-0,094 a 0,098)	0,967
Uso de IECA/BRA	-0,121 (-0,217 a -0,025)	0,015
Diabetes	0,071 (-0,025 a 0,167)	0,153
AOS	0,027 (-0,073 a 0,127)	0,602
Status tabágico: atual/não registrado	0,030 (-0,064 a 0,124)	0,529
Dexametasona: sim	0,041 (-0,063 a 0,145)	0,444
Remdesivir: sim	0,101 (-0,003 a 0,205)	0,055

As variáveis preditoras foram codificadas da maneira seguinte: feminino = 0, masculino = 1; controle = 0, em uso IECA/BRA = 1; sem diabetes = 0, diabetes = 1; sem AOS = 0, AOS = 1; status tabágico: nunca/ex-fumante = 0, atual fumante/não registrado = 1; não tomou dexametasona = 0, tomou dexametasona = 1; não tomou remdesivir = 0, tomou remdesivir = 1. Código 0 considerado como referência. Os números entre parênteses são intervalos de confiança de 95%. AOS: apneia obstrutiva do sono; BRA: bloqueador do receptor de angiotensina; IECA: inibidor da enzima conversora de angiotensina; IMC: índice de massa corporal.

### Análise de sensibilidade usando o status tabágico na mortalidade e no tempo de internação hospitalar

Devido à falta de dados sobre o status tabágico em todos os pacientes, foi realizada uma análise de sensibilidade usando o status tabágico para avaliar a confiabilidade dos resultados do estudo. Havia 33 pacientes (n = 23 em IECA/BRA e n = 10 no grupo de comparação) com status tabágico desconhecido. O cenário mais extremo seria considerar todos os pacientes com status desconhecido como fumantes. Nesse cenário, observamos chances diminuídas de mortalidade em fumantes em uso de IECA/BRA ( Tabela 3 ). Após remover da análise os pacientes com dados ausentes sobre o estado tabágico, o efeito protetor do uso de IECA/BRA na mortalidade e no tempo de internação foi preservado ( Tabelas Suplementares 1 e 2 ).

### Associações ajustadas entre o uso de IECA/BRA e outras complicações da COVID-19

A associação entre o uso de IECA/BRA e outras complicações da COVID-19 foi avaliada após ajuste para sexo, idade, IMC, histórico de tabagismo e diabetes. Em relação ao grupo de comparação, os pacientes do grupo IECA/BRA manifestaram tendências não significativas de menor associação com internação na UTI, ventilação mecânica, uso de vasopressores e oxigênio suplementar do que o grupo de comparação. As Tabelas Suplementares 3 , 4 , 5 e 6 resumem as análises de regressão logística para esses desfechos, respectivamente.

## Discussão

Até onde sabemos, este é o primeiro estudo dedicado a avaliar o prognóstico agudo de COVID-19 em pacientes com hipertensão relacionada ao sobrepeso/obesidade que estavam tomando IECA/BRA antes da internação hospitalar. Observou-se associação significativa entre a exposição aos IECA/BRA e a redução da mortalidade por COVID-19, mesmo após ajuste estatístico. Embora tendências favoráveis tenham sido observadas no grupo IECA/BRA, nenhuma diferença significativa foi demonstrada nas taxas de internação em UTI, ventilação mecânica e uso de vasopressores.

Atualmente, a análise de nossos dados ecoa as recomendações de várias sociedades profissionais de que os médicos clínicos não devem descontinuar os IECA/BRA dos pacientes antes ou depois da COVID-19, a menos que seja clinicamente indicado na doença grave.^6 , 18^ Nossa análise confirma os achados de um estudo recente relatando associações entre o uso de IECA/BRA e a redução da gravidade e mortalidade por COVID-19.^6^ Zhang et al.^6^ conduziram um estudo retrospectivo multicêntrico com 1.128 pacientes hospitalizados com COVID-19 e hipertensão mostrando que o uso de IECA/BRA foi associado a um menor risco de mortalidade por todas as causas ( *hazard ratio* ajustada 0,42; p = 0,03).^6^ Embora este estudo indique uma associação entre o uso de IECA/BRA e menor mortalidade, o número de pacientes com sobrepeso/obesidade não foi relatado. É, no entanto, provável que houvesse vários pacientes com sobrepeso/obesidade, dado o número de pacientes com comorbidades relacionadas ao sobrepeso/obesidade, como diabetes mellitus, doença arterial coronariana e doença hepática. Adicionalmente, nossos dados sugerem que o uso de IECA/BRA está associado a uma diminuição do tempo de internação. Essa tendência está de acordo com um recente estudo observacional multicêntrico de Braude et al. (2018) que mostrou redução no tempo de internação em pacientes em uso de IECA/BRA.^18^ Novamente, os autores não relataram o número de pacientes com sobrepeso/obesidade em sua análise, embora seja provável que vários estivessem com sobrepeso/obesidade, dado que o estudo consistia principalmente de uma população idosa e doente.

O uso de IECA/BRA entre pacientes com COVID-19 e hipertensão relacionada à obesidade tem sido fonte de especulação entre os especialistas em hipertensão.^6^ É comumente reconhecido que há um desequilíbrio do SRAA em pacientes com obesidade. Existe uma superexpressão dos receptores da angiotensina I (AT1R) e dos receptores da angiotensina II no nível do tecido adiposo e no nível sistêmico.^18^ Originalmente, pensava-se que esse mecanismo contribuía para uma lesão pulmonar aumentada em resposta ao vírus em pacientes com COVID-19 e sobrepeso/obesidade. No entanto, observou-se que pacientes com sobrepeso/obesidade estavam, de fato, protegidos da mortalidade. Uma possível explicação sugerida por vários autores é que o tratamento com IECA/BRA no contexto de infecção concomitante com SARS-CoV-2 pode alterar o equilíbrio fisiológico entre o eixo ECA/ang II/AT1R para o eixo ECA2/ang 1-7/receptor MAS. O metabólito ang 1-7 pode estar atuando no receptor MAS para desempenhar um papel na proteção cardiovascular, um efeito anti-inflamatório global e potencialmente até atenuar a lesão pulmonar.^19^ Embora tenha sido originalmente postulado que a regulação positiva da ECA2 pelo tratamento com IECA/BRA aumentava a infecção por SARS-CoV-2, ela pode estar desempenhando um papel protetor em pacientes ao regular positivamente a produção de espécies de angiotensina 1-7 e bloqueando a indução de citocinas pró-inflamatórias. Esse efeito anti-inflamatório global pode ser ainda mais ampliado em pacientes com obesidade, dado um número aumentado de células que expressam ECA2 e, consequentemente, uma quantidade maior de ECA2.^20^ Mais pesquisas mecanísticas e translacionais são necessárias para estudar o efeito de IECA/BRA na expressão pulmonar de ECA2.^21 - 23^

Embora houvesse mais homens e uma taxa maior de comorbidades como AOS e diabetes no grupo IECA/BRA, uma taxa de mortalidade menor foi observada nesse grupo em relação ao grupo de comparação. Isso fortalece os nossos resultados em relação ao efeito protetor de IECA/BRA, já que os homens são considerados de maior risco para morte relacionada à COVID-19.^24^ Uma ampla gama de variáveis biopsicossociais deve ser considerada para validar essa associação. Relata-se que os andrógenos aumentam a concentração e a atividade da renina plasmática, levando a níveis mais altos de proteína e mRNA do angiotensinogênio.^24^ O efeito a jusante disso é a vasoconstrição sistêmica e a COVID-19 mais grave. Assim, os IECA/BRA podem potencialmente ser mais benéficos na população masculina. Adicionalmente, não houve associação entre AOS e mortalidade, e não houve interação entre o uso de IECA/BRA e AOS na mortalidade. Portanto, não foram realizados ajustes para uso de pressão positiva contínua nas vias aéreas, apesar de uma diferença significativa entre os dois grupos no uso de pressão positiva contínua nas vias aéreas.

Nossos resultados também sugerem que o tratamento com remdesivir está associado à hospitalização prolongada (p = 0,055, IC 95% 0,003 a 0,205), mas não à redução da mortalidade (p = 0,345, IC 95% 0,755 a 2,233). Este achado está de acordo com um estudo em pacientes adultos com COVID-19 internados em 123 hospitais da Veterans Health Administration de 1º de maio a 8 de outubro de 2020, que também mostrou que o tratamento com remdesivir não foi associado a melhor sobrevida, mas sim a internações hospitalares mais longas.^25^

O presente estudo tem várias limitações. Primeiro, não foi possível estabelecer causalidade entre a exposição a IECA/BRA e a gravidade e mortalidade por COVID-19 devido à limitação inerente do desenho de estudo retrospectivo. Em segundo lugar, esses dados foram acumulados em um único centro e o tamanho da amostra foi pequeno; portanto, esses resultados podem não ser generalizáveis. O intervalo de confiança para mortalidade foi grande, provavelmente indicativo do pequeno tamanho da amostra, e isso limitou o poder de nosso estudo. Apesar do pequeno tamanho da amostra, no entanto, uma significância estatística para desfechos clinicamente importantes ainda foi observada. Em terceiro lugar, embora modelos estatísticos tenham sido usados para ajustar possíveis vieses, os resultados não foram ajustados para etnia, o que pode ter influenciado os resultados. Em quarto lugar, o uso real de IECA/BRA não pôde ser verificado a partir dos registros médicos, que apenas relatam se o paciente recebeu ou não uma prescrição para esses medicamentos. Em quinto lugar, alguns hábitos de vida, como uso de álcool e adesão a medicamentos/nível de controle da pressão arterial, não estavam disponíveis ou não foram coletados neste estudo. Isso poderia potencialmente confundir algumas das associações observadas neste estudo. Em sexto lugar, com o objetivo de preservar o poder do estudo, as análises consideraram usuários de IECA e BRA combinados para estimativas dos desfechos. No entanto, quando analisados de forma independente, IECA e BRA também foram associados à redução da mortalidade (OR 0,557, IC 95% 0,315 a 0,987, p = 0,045 e OR 0,388, IC 95% 0,189 a 0,797, p = 0,01; respectivamente).

É importante ressaltar que abordamos a limitação da falta de dados em relação ao status tabágico entre os registros médicos por meio da realização de uma análise de sensibilidade. A análise considerando todos os pacientes com status desconhecido como fumantes mostrou que os fumantes tinham uma chance significativamente maior de mortalidade. Esse efeito, no entanto, desapareceu após a remoção dos pacientes que não tinham dados sobre o status tabágico. A observação de que pacientes fumantes apresentam maior mortalidade é corroborada por vários estudos.^26^ Recentemente, um estudo de Lowe et al. (2021) mostrou que a exposição cumulativa à fumaça de cigarro foi um fator de risco independente para internação hospitalar e mortalidade relacionada à COVID-19. Além disso, o efeito do tabagismo na mortalidade exibiu uma relação dose-dependente, porque pacientes com mais maços-ano exibiram maiores chances de morte relacionada à COVID-19.^27^

## Conclusão

Estes resultados sugerem que pacientes hospitalizados com COVID-19 e hipertensão relacionada ao sobrepeso/obesidade que receberam IECA/BRA antes da internação hospitalar apresentam menor tempo de internação e mortalidade relacionada à COVID-19 em comparação com aqueles que não estavam tomando IECA/BRA. Essa observação pode trazer implicações clínicas importantes em termos de tratamento da COVID-19 nesse grupo único de pacientes com sobrepeso/obesidade.

### Materiais suplementares

As Tabelas Suplementares S1 a S6 indexam e contém os seguintes dados adicionais: S1: Preditores de mortalidade entre pacientes com COVID-19 e hipertensão relacionada ao sobrepeso/obesidade nos grupos IECA/BRA e comparação, com a remoção do status tabágico desconhecido. S2: Preditores de tempo de internação hospitalar entre pacientes com COVID-19 e hipertensão relacionada ao sobrepeso/obesidade nos grupos IECA/BRA e comparação, com a remoção do status tabágico desconhecido. S3: Preditores de terapia intensiva entre pacientes com COVID-19 e hipertensão relacionada ao sobrepeso/obesidade nos grupos IECA/BRA e comparação. S4: Preditores de ventilação mecânica entre pacientes com COVID-19 e hipertensão relacionada ao sobrepeso/obesidade nos grupos IECA/BRA e comparação. S5: Preditores do uso de vasopressores entre pacientes com COVID-19 e hipertensão relacionada ao sobrepeso/obesidade nos grupos IECA/BRA e comparação. S6: Preditores de uso de oxigênio suplementar entre pacientes com COVID-19 e hipertensão relacionada ao sobrepeso/obesidade nos grupos IECA/BRA e comparação.

### * Material suplementar

Para informação adicional, por favor, clique aqui .
