# Supplementary material for: Associação entre o Antagonismo do Sistema Renina-Angiotensina-Aldosterona e a Mortalidade Relacionada à COVID-19 em Pacientes com Hipertensão Relacionada ao Sobrepeso/Obesidade: um Estudo Retrospectivo de Coorte
Source: Arq Bras Cardiol. 2023 Mar 24;120(4):e20220277. [Article in Portuguese] doi: 10.36660/abc.20220277 (PMC10263421; doi:10.36660/abc.20220277)
Supplement: Translation

# Translation: Association between RAAS Antagonism and COVID-19–related Mortality in Patients with Overweight/Obesity-related Hypertension: A Retrospective Cohort Study

**Authors:** Elham Shams, Vijayvardhan Kamalumpundi, Linhai Cheng, Adeyinka Taiwo, Amal Shibli-Rahhal, Ayotunde O. Dokun, Marcelo L.G. Correia

**Affiliations:** 1 Division of Endocrinology and Metabolism Department of Internal Medicine University of lowa USA Division of Endocrinology and Metabolism, Department of Internal Medicine, University of lowa – USA; 2 Carver College of Medicine University of Iowa USA Carver College of Medicine, University of Iowa - USA

**Keywords:** Angiotensin Receptor Antagonists, Angiotensin-Converting Enzyme Inhibitors, COVID-19, Obesity, Hypertension

## Abstract

**Background:**

Angiotensin receptor blockers (ARB) and angiotensin-converting enzyme inhibitors (ACEI) increase the expression of ACE2, which is a receptor for entry of SARS-CoV-2 into cells. Though evidence suggests that ARB/ACEI are safe among the general population with COVID-19, their safety in patients with overweight/obesity-related hypertension deserves further evaluation.

**Objective:**

We assessed the association between ARB/ACEI use and COVID-19 severity in patients with overweight/obesity-related hypertension.

**Methods:**

This study included 439 adult patients with overweight/obesity (body mass index ≥ 25 kg/m^2^) and hypertension, diagnosed with COVID-19 and admitted to University of Iowa Hospitals and Clinic from March 1 to December 7, 2020. Mortality and severity of COVID-19 were evaluated based on length of stay in hospital, intensive care unit admission, use of supplemental oxygen, mechanical ventilation, and vasopressors. Multivariable logistic regression was used to examine the associations of ARB/ACEI use with mortality and other markers of COVID-19 severity, with a two-sided alpha set at 0.05.

**Results:**

Exposure to ARB (n = 91) and ACEI (n = 149) before hospitalization was significantly associated with lower mortality (odds ratio [OR] = 0.362, 95% confidence interval [CI] 0.149 to 0.880, p = 0.025) and a shorter length of stay (95% CI −0.217 to −0.025, p = 0.015). Additionally, patients using ARB/ACEI showed a non-significant trend toward lower intensive care unit admission (OR = 0.727, 95% CI 0.485 to 1.090, p = 0.123), use of supplemental oxygen (OR = 0.929, 95% CI 0.608 to 1.421, p = 0.734), mechanical ventilation (OR = 0.728, 95% CI 0.457 to 1.161, p = 0.182), and vasopressors (OR = 0.677, 95% CI 0.430 to 1.067, p = 0.093).

**Conclusion:**

Results suggest that hospitalized patients with COVID-19 and overweight/obesity-related hypertension who were prescribed ARB/ACEI before admission to the hospital exhibit lower mortality and less severe COVID-19 than those who were not taking ARB/ACEI. The results also suggest that exposure to ARB/ACEI may protect patients with overweight/obesity-related hypertension from severe COVID-19 and death.

**Central Illustration:** : Association between RAAS Antagonism and COVID-19–related Mortality in Patients with Overweight/Obesity-related Hypertension: A Retrospective Cohort Study

ACEI/ARB: angiotensin-converting enzyme inhibitors/angiotensin receptor blockers; BMI: body mass index.

## Introduction

The coronavirus disease 2019 (COVID-19) pandemic was caused by a novel, positive sense, single-stranded RNA virus called severe acute respiratory syndrome coronavirus 2 (SARS-CoV-2). To date, COVID-19 has claimed over 6.3 million lives globally, with the United States leading the world in the absolute number of deaths.^1 - 3^A growing body of evidence suggests that older age (≥ 65 years) and comorbidities such as hypertension, diabetes, and obesity are independent risk factors for more severe COVID-19 as compared to the general population.^2 , 4^

The mechanism of viral entry by SARS-CoV-2 has been well characterized.^2 , 3 , 5^The novel virus exploits the cell-membrane bound aminopeptidase angiotensin-converting enzyme 2 (ACE2) for viral entry and replication.^2^Mechanistic evidence in select experimental mouse models has shown that ACE2 expression is upregulated with angiotensin receptor blocker/angiotensin-converting enzyme inhibitor (ARB/ACEI) administration.^5^This mechanism initially drove many clinicians and researchers to postulate that ARB/ACEI, both of which block the renin-angiotensin-aldosterone system (RAAS), may increase the severity of COVID-19 infection by promoting SARS-CoV-2 binding and cellular entry. Indeed, current clinical data overwhelmingly suggest that ARB/ACEI do not increase COVID-19 severity and mortality; however, to date, no study has been conducted exclusively in a population with overweight/obesity.

It is well known that patients with obesity have augmented RAAS activity, with adipocytes in visceral adipose tissue highly expressing ACE2.^6^In addition, given the metabolic dysregulation, proinflammatory state, and higher rates of thrombosis, obesity is known to increase the risk of severe COVID-19 and COVID-19–related mortality. In a recent study among 6,760 hospitalized health care personnel with COVID-19 in 14 states in the US, obesity (72.5%) was found to be the most prevalent comorbidity.^4^Similarly, in a single-center, retrospective cohort study conducted in France with 124 patients with COVID-19 admitted to intensive care, the risk for invasive mechanical ventilation was nearly 7-fold higher for those with a body mass index (BMI) >35 kg/m^2^.^7^In addition to obesity, hypertension and other cardiovascular comorbidities are the most common comorbid diseases in patients with COVID-19.^8^Several multi-center observational studies and population-wide studies have demonstrated that patients with hypertension experience more severe COVID-19 and higher mortality.^8 - 10^

Several studies have shown that higher BMI has been related to a higher prevalence of cardiovascular disorders such as hypertension, stroke, myocardial infarction, and ischemic heart disease. Furthermore, most patients with severe COVID-19 who were hospitalized suffered from comorbidities related to excess adiposity, such as diabetes and cardiovascular disorders,^11^which is also in line with previous reports from H1N1 influenza outbreaks.^12^In a retrospective study of 1965 patients with diabetes, in which 726 (36.9%) patients were overweight and 805 (41.0%) had obesity, the authors reported that these conditions were associated with poor prognosis in patients with type 2 diabetes hospitalized for COVID-19.^13^

The RAAS consists of an enzymatic cascade responsible for blood pressure control by maintaining fluid and electrolyte balance and preserving systemic vascular resistance.^3^The cascade starts when plasma angiotensinogen is cleaved by renin, into angiotensin I (ang I). Angiotensin-converting enzyme then catalyzes ang I into angiotensin II (ang II).^1^ACE2 uses ang II as a substrate and produces ang (1-7). Ang (1-7) is a metabolically active hormone that acts at the MAS receptor to lower blood pressure and has anti-inflammatory and anti-fibrotic properties. ACEIs inhibit the formation of ang II from ang I. This event leads to conversion of ang I to a similar hormone, ang 1-9. Ang 1-9 is rapidly converted to ang 1-7 by ACE2. ARBs prevent ang II from binding to its receptor; thus, ARBs inhibit the effect of ang II.^2 , 3^

ARB/ACEIs are used extensively in patients with hypertension, other cardiovascular diseases, and diabetes to treat high blood pressure, heart failure, chronic kidney disease, and many other diseases.^14^These diseases are often comorbid conditions with obesity; therefore, clinical data about the usage of these medications in patients with overweight/obesity-related hypertension is warranted. To add to the current evidence base regarding the clinical outcomes of this demographic, a retrospective cohort study was conducted to investigate whether there is an association between exposure to ARB/ACEI prior to admission and the severity of COVID-19 among patients with overweight/obesity-related hypertension.

## Methods

### Data source and patients

This was a single-center, retrospective cohort study conducted on patients diagnosed with COVID-19 who were hospitalized at the University of Iowa Hospitals and Clinic (UIHC). Electronic medical records at the University of Iowa (Epic 2018 version UI 2, Epic Systems Corporation, Verona, WI, USA) were used to identify all patients who (1) were ≥ 18 years of age, (2) had a BMI of ≥ 25 kg/m^2^, and (3) had a diagnosis of hypertension, admitted to UIHC between March 1 and December 7, 2020 with COVID-19. The electronic medical records contain complete demographic, clinical, laboratory, and medication data for all patients seen at our medical center. Patients with an incomplete electronic medical record and diagnoses of gestational, pulmonary, portal, renal, and secondary hypertension were excluded. Patients who stopped taking medications for the treatment of hypertension for any reason (e.g., an increase of creatinine level, unable to orally take medicines due to mechanical ventilation, etc.) during hospitalization were included in the study. In addition, patients taking ARB/ACEI for any other indication other than hypertension (e.g., diabetes-related microalbuminuria) were excluded.

### Data elements

Data elements included: age, BMI, sex, smoking/alcohol history, comorbidities (e.g. diabetes and obstructive sleep apnea [OSA]), complications during hospitalization (e.g. pancreatitis, stroke, acute respiratory distress syndrome, hepatitis, etc.), treatments for COVID-19 (e.g. remdesivir, dexamethasone, azithromycin, chloroquine, hydroxychloroquine), ventilatory support (e.g. supplemental oxygen, extracorporeal membrane oxygenation, invasive ventilation, non-invasive ventilation), intensive care unit (ICU) admission, use of vasopressors, in-hospital mortality, and length of hospital stay. COVID-19 diagnosis was determined by positive test using nasopharyngeal swabs with rare use of oropharyngeal swabs and sputum. All samples were collected in non-activating viral transport media from various manufacturers with high-quality flocked swabs (Center for Disease Control 2019-nCoV Real-Time RT-PCR Diagnostic Panel, Center for Disease Control Emergency Operations Center, Atlanta, GA, USA; TaqPATH COVID-19 Combo kit, Cat#: A47814, Thermo Fisher Scientific Inc., Waltham, MA, USA.). ARB/ACEI use was defined as use of these drugs at the time of admission that was halted during hospitalization. According to their antihypertensive medication, patients were divided into 2 subgroups, those who had a prescription for ARB/ACEI before admission to the hospital and those who did not have a prescription for ARB/ACEI (i.e., the comparison group). All data were extracted from the patients’ electronic medical records by 3 investigators (ES, VK, LC) using a standardized data collection form that was then cross-checked by 4 investigators (ES, VK, LC, MC).

### Statistical analysis

Statistical analysis was performed with SPSS software (IBM SPSS Statistics for Windows, Version 27.0. Armonk, NY, USA). Descriptive statistics were used to summarize patient demographics and clinical characteristics. The Kolmogorov-Smirnov test was used to assess the distribution of variables. Categorical variables were presented as numbers and percentages and compared using the chi-square test. Continuous variables were presented as mean and standard deviation. Independent sample t-tests were used to compare continuous variables. Binary logistic regression analysis was used to analyze binary dependent outcomes such as mortality, ICU admission, use of supplemental oxygen_,_mechanical ventilation, and vasopressors. All models included use of ARB/ACEI as an explanatory variable and were adjusted for a set of covariates, determined *a priori* , that could confound the association between the use of ARB/ACEI and illness severity. These independent covariates included age, sex, BMI, smoking history, OSA, diabetes, and use of remdesivir and dexamethasone. Tests for interaction were conducted between independent variables. Multivariate linear regression models were used for continuous outcomes such as length of stay. Results were presented as odds ratios (ORs) with 95% confidence interval (95% CI) and adjusted p value. P values below a two-sided alpha of 0.05 were considered statistically significant.

By the time this study was conducted, the Infectious Disease Society of America (IDSA) had recommended remdesivir for patients in the early stage of COVID-19 to reduce viral replication and dexamethasone for patients in later stages of the illness to reduce the production of pro-inflammatory cytokines.^15^There is strong evidence that remdesivir and dexamethasone can change the course of COVID-19.^16 , 17^Therefore, these variables were added to the model as post-treatment covariates to determine if there was an effect of ARB/ACEI exposure independent of remdesivir and/or dexamethasone effects. Data on antibiotics other than azithromycin were not collected. In addition, data regarding the use of hydroxychloroquine/chloroquine and azithromycin plus hydroxychloroquine to reduce COVID-19–related mortality was considered inconsistent and thus not added to the model, but were nevertheless collected and included in descriptive statistics.

## Results

### Baseline characteristics

The study identified 946 patients with confirmed COVID-19 who were admitted to UIHC. After applying the exclusion criteria, 439 patients were included in the final analysis ( Figure 1 ). This cohort was subsequently grouped into the ARB/ACEI group (n = 240) and comparison group (n = 199). Patient demographics are shown in Table 1 , and their outcomes, and complications are shown in Table 2 . The mean ages were similar across both groups. Compared to the comparison group, patients who were using ARB/ACEI had a significantly higher proportion of men and a higher rate of diabetes mellitus. The rate of OSA in patients who were using ARB/ACEI was higher than in the comparison group, but this difference was not statistically significant (p = 0.186). In addition, there were more current smokers in the comparison group than the ARB/ACEI group.

**Figure 1:** – Study Flowchart. Inclusion criteria: ≥ 18 years of age, diagnosed with COVID-19, hypertension, and BMI ≥ 25 kg/m2. ACEI: angiotensin-converting enzyme inhibitor; ARB: angiotensin receptor blocker; COVID-19: coronavirus disease 2019; HTN: hypertension.

**Table 1:** – Demographics of patients with overweight/obesity-related hypertension among ARB/ACEI and comparison groups

	ARB/ACEI (n = 240)	Comparison (n = 199)	p value
**Demographics**			
Age, years (SD)	61.9 (13.3)	63.2 (15.1)	0.358
BMI (SD)	34.6 (8.4)	34.3 (7.9)	0.765
**Sex**			
Male (%)	181 (75.4)	116 (58.2)	<0.001
**Chronic comorbidities, N (%)**			
Diabetes	157 (65.4)	88 (44.2)	<0.001
OSA	74 (30.8)	50 (25.1)	0.186
CPAP usage	49 (20.4)	23 (11.5)	0.013
**Smoking, N (%)**			
Never smoker	100 (41.6)	87 (43.7)	
Former smoker	107 (44.5)	92 (46.2)	
Current smoker	10 (4.1)	10 (5.0)	0.289^*^
**Treatments, N (%)**			
Remdesivir	83 (34.5)	72 (36.1)	0.727
Convalescent plasma	33 (13.7)	31 (15.5)	0.589
Dexamethasone	123 (51.2)	107 (53.7)	0.599
Chloroquine	0 (0)	0 (0)	--
Azithromycin	33 (13.7)	24 (12.0)	0.6
Hydroxychloroquine	2 (0.8)	1 (0.5)	1

P values were calculated from independent sample t-tests and chi square tests, where appropriate. *The p value for smokers was calculated between smoker (current and unknown) and non-smoker (former) categories. ACEI: angiotensin-converting enzyme inhibitor; ARB: angiotensin receptor blocker; BMI: body mass index; CPAP: continuous positive airway pressure; OSA: obstructive sleep apnea; SD: standard deviation.

**Table 2:** – Outcomes and complications of patients with overweight/obesity-related hypertension among ARB/ACEI and comparison groups

	ARB/ACEI (n = 240)	Comparison (n = 199)	p value
**Outcome, N (%)**			
Supplemental oxygen	158 (65.8)	130 (65.3)	0.911
Non-invasive ventilation	125 (52.0)	106 (53.2)	0.805
ECMO	3 (1.2)	6 (3.0)	0.311
Intensive care	95 (39.5)	86 (43.2)	0.441
Mechanical ventilation	54 (22.5)	51 (25.6)	0.444
Vasopressors	57 (23.7)	56 (28.1)	0.295
Mortality	45 (18.7)	51 (25.6)	0.083
**Complications, N (%)**			
Respiratory disease	78 (32.5)	54 (27.1)	0.222
Kidney and urinary tract diseases	34 (14.1)	21 (10.5)	0.255
Liver disease	8 (3.3)	3 (1.5)	0.359
Cardiovascular disease	13 (5.4)	15 (7.5)	0.365
Pancreatic disease	2 (0.8)	1 (0.5)	1
Hematologic disease	9 (3.7)	7 (3.5)	0.897
Septic shock	10 (4.1)	6 (3.0)	0.522
Elevated blood sugar	2 (0.8)	2 (1.0)	1
Acute encephalopathy	8 (3.3)	7 (3.5)	0.916

P values were calculated from independent sample t-tests and chi square test, where appropriate. ACEI: angiotensin-converting enzyme inhibitor; ARB: angiotensin receptor blocker; ECMO: extracorporeal membrane oxygenation.

### Medications administered in hospital

Among the medications administered for COVID-19 treatment, fewer patients received dexamethasone in the ARB/ACEI group as opposed to the comparison group, although this difference was not statistically significant ( Table 1 ).

### Unadjusted associations between ARB/ACEI and primary outcomes

The unadjusted mortality rate was lower in the ARB/ACEI group than comparison group (18.7% versus 25.6%, p = 0.083). Furthermore, compared with the comparison group, patients in the ARB/ACEI group had a lower ICU admission rate and less use of mechanical ventilation and vasopressors. Overall, unadjusted for other covariates, the illness severity of COVID-19 for patients in the comparison group was worse than in the ARB/ACEI group.

### Adjusted associations between ARB/ACEI use and mortality

After statistical adjustment by age, sex, BMI, smoking history, OSA, diabetes, and usage of dexamethasone and remdesivir, the ARB/ACEI group had significantly lower odds of mortality than patients in the comparison group ( Table 3 ). In addition, results from the adjusted models showed that older age and male sex were associated with increased odds of COVID-19–related mortality. The interaction between use of ARB/ACEI and diabetes was not significant. Furthermore, there was no association between OSA and mortality, and no interaction between ARB/ACEI use and OSA on mortality.

**Table 3:** – Logistic regression analysis of predictors of mortality between patients with COVID-19 e overweight/obesity-related hypertension in ARB/ACEI and comparison groups

Variables	Odds ratio	p value
Age	1.030 (1.010–1.051)	0.003
BMI	0.985 (0.950–1.022)	0.416
Sex (male)	2.029 (1.149–3.584)	0.015
Using ARB/ACEI	0.362 (0.149–0.880)	0.025
Diabetes	1.701 (0.860–3.364)	0.127
ACEI or ARB by diabetes	1.569 (0.532– 4.624)	0.414
OSA	1.237 (0.710–2.157)	0.453
Smoking status: current/not on file	2.935 (1.520–5.665)	0.001
Dexamethasone: yes	0.970 (0.567–1.659)	0.910
Remdesivir: yes	1.298 (0.755–2.233)	0.345

Predictor variables were coded as follows: female = 0, male = 1; control = 0, using ACEI/ARB = 1; without diabetes = 0, diabetes = 1; without OSA = 0, OSA = 1; smoking status: never/former = 0, current/not on file = 1; did not take dexamethasone = 0, took dexamethasone = 1; did not take remdesivir = 0, took remdesivir = 1; survival = 0, mortality = 1. Code 0 considered as reference. Numbers in parentheses are 95% confidence intervals. ACEI: angiotensin-converting enzyme inhibitor; ARB: angiotensin receptor blocker; BMI: body mass index; OSA: obstructive sleep apnea.

### Adjusted associations between ARB/ACEI use and length of stay

All parameters met the necessary assumptions for linear regression. The multivariate linear regression model showed that patients in the ARB/ACEI group had a shorter length of stay after adjustment for age, sex, BMI, diabetes, OSA, smoking status, and use of dexamethasone and remdesivir ( Table 4 ).

**Table 4:** – Logistic regression analysis of predictors of length of stay in hospital between patients with COVID-19 and overweight/obesity-related hypertension in ARB/ACEI and comparison groups

Variables	Standardized B	p value
Age	0.002 (-0.096 a 0.100)	0.962
BMI	-0.056 (-0.160 a 0.048)	0.288
Sex (male)	0.002 (-0.094 a 0.098)	0.967
Using ARB/ACEI	-0.121 (-0.217 a -0.025)	0.015
Diabetes	0.071 (-0.025 a 0.167)	0.153
OSA	0.027 (-0.073 a 0.127)	0.602
Smoking status: current/not on file	0.030 (-0.064 a 0.124)	0.529
Dexamethasone: yes	0.041 (-0.063 a 0.145)	0.444
Remdesivir: yes	0.101 (-0.003 a 0.205)	0.055

Predictor variables were coded as follows: female = 0, male = 1; control = 0, using ACEI/ARB = 1; without diabetes = 0, diabetes = 1; without OSA = 0, OSA = 1; smoking status: never/former = 0, current/not on file = 1; did not take dexamethasone = 0, took dexamethasone = 1; did not take remdesivir = 0, took remdesivir = 1. Code 0 considered as reference. Numbers in parentheses are 95% confidence intervals. ACEI: angiotensin-converting enzyme inhibitor; ARB: angiotensin receptor blocker; BMI: body mass index; OSA: obstructive sleep apnea.

### Sensitivity analysis using smoking status on mortality and length of stay

Due to a lack of data regarding smoking status on all patients, a sensitivity analysis was conducted using smoking status to assess the reliability of the study results. There were 33 patients (n = 23 in ARB/ACEI and n = 10 in the comparison group) with smoking status unknown. The most extreme scenario is considering all patients with unknown smoking status as smokers. In this scenario, we observed decreased odds of mortality in smokers using ARB/ACEI ( Table 3 ). After removing the patients with missing data regarding smoking status from the analysis, the protective effect of ARB/ACEI use on mortality and length of stay was preserved ( Supplemental Tables 1 and 2 ).

### Adjusted associations between ARB/ACEI use and other COVID-19 complications

The association between the use of ARB/ACEI and other COVID-19 complications was evaluated after adjusting for sex, age, BMI, smoking history, and diabetes. Compared to the comparison group, patients in the ARB/ACEI group manifested non-significant trends toward lower association with ICU admission, mechanical ventilation, usage of vasopressors, and supplemental oxygen than the comparison group. Supplemental Tables 3 , 4 , 5 , and 6 summarize logistic regression analyses of these outcomes respectively.

## Discussion

To our knowledge, this is the first dedicated study to evaluate the acute prognosis of COVID-19 in patients with overweight/obesity-related hypertension who were taking ARB/ACEI prior to hospital admission. A significant association was observed between the exposure to ARB/ACEI and reduced mortality from COVID-19, even after statistical adjustment. While favorable trends were observed in the ARB/ACEI group, no significant differences were demonstrated in the rates of ICU admission, mechanical ventilation, and use of vasopressors.

At present, analysis of our data echoes recommendations from several professional societies, that clinicians should not discontinue patients’ ARB/ACEI medications before or after COVID-19, unless clinically indicated in severe disease.^6 , 18^Our analysis confirms the findings of a recent study reporting associations between the use of ARB/ACEI and reduced COVID-19 severity and mortality.^6^Zhang et al.^6^conducted a multi-center, retrospective study of 1128 hospitalized patients with COVID-19 and hypertension, and showed that use of ARB/ACEI was associated with a lower risk of all-cause mortality (adjusted hazard ratio 0.42; p = 0.03).^6^Though this study pointed to an association between ARB/ACEI use and lower mortality, it did not report the number of patients with overweight/obesity in their analyses. It is, however, likely that there were several patients with overweight/obesity in this study given the number of patients with overweight/obesity-related comorbidities like diabetes mellitus, coronary artery disease, and liver disease. In addition, our data suggest that the use of ARB/ACEI is associated with a decreased length of stay. This trend is concordant with a recent multi-center, observational study by Braude et al. (2018) that showed a reduction in the length of stay in patients using ARB/ACEI.^18^Again, the authors did not report the number of patients with overweight/obesity in their analysis, though it is likely that several were with overweight/obesity given that the study primarily consisted of an elderly and diseased population.

The use of ARB/ACEI among patients with COVID-19 and obesity-related hypertension has been a source of speculation among hypertension specialists.^6^It is commonly recognized that there is an imbalance of RAAS in patients with obesity. There is an overexpression of angiotensin I receptor (AT1R) and angiotensin II receptors at the level of the adipose tissue and at a systemic level.^18^This mechanism was originally thought to contribute to an augmented lung injury in response to the virus in patients with COVID-19 and overweight/obesity. However, it has been observed that patients with overweight/obesity were, in fact, protected from mortality. A possible explanation that has been suggested by several authors is that ARB/ACEI treatment in the context of concurrent infection with SARS-CoV-2 may shift the physiologic balance between the ACE/ang II/AT1R axis to the ACE2/ang 1-7/MAS receptor axis. The metabolite ang 1-7 could be acting on the MAS receptor to play a role in cardiovascular protection, a global anti-inflammatory effect and potentially even attenuating lung injury.^19^Though upregulation of ACE2 by ARB/ACEI treatment was originally posited to increase SARS-CoV-2 infection, it may be serving a protective role in patients by upregulating production of angiotensin 1-7 species and abrogating the induction of pro-inflammatory cytokines. This global anti-inflammatory effect could be even more magnified in patients with obesity, given an increased number of ACE2-expressing cells, and, subsequently, larger amount of ACE2.^20^Further mechanistic and translational research is required to study the effect of ARB/ACEI on pulmonary ACE2 expression.^21 - 23^

Interestingly, even though there were more males and a higher rate of comorbidities such as OSA and diabetes in the ARB/ACEI group, a lower mortality rate was observed within this group as compared to the comparison one. This lends confidence to our results regarding the protective effect of ARB/ACEI, as males are regarded to be at a higher risk of COVID-19–related death.^24^A wide range of biopsychosocial variables must be considered to validate this association; androgens are reported to increase plasma renin concentration and activity leading to higher levels of angiotensinogen mRNA and protein.^24^The downstream effect of this is systemic vasoconstriction and more severe COVID-19. Thus, ARB/ACEI may potentially be more beneficial in the male population. In addition, there was no association between OSA and mortality, and no interaction between ARB/ACEI exposure and OSA on mortality. Therefore, no adjustments for continuous positive airway pressure usage were conducted despite a significant difference between both groups in continuous positive airway pressure usage.

Our results also suggest that treatment with remdesivir is associated with prolonged hospitalization (p = 0.055, 95% CI 0.003 to 0.205), but not with reduced mortality (p = 0.345, 95% CI 0.755 to 2.233). This finding is in line with a study in adult patients with COVID-19 admitted to 123 Veterans Health Administration hospitals from May 1 to October 8, 2020, which also showed that remdesivir treatment was not associated with improved survival, but instead with longer hospital stays.^25^

The current study has several limitations. First, it could not establish causality between the exposure to ARB/ACEI and the severity and mortality in COVID-19 because of the inherent limitation of a retrospective study design. Second, these data were accrued at a single center, and the sample size was small; therefore, these results may not be generalizable. The confidence interval for mortality was large, likely indicative of the small sample size, and this limited the power of our study. Despite the small sample size, however, a statistical significance for clinically important outcomes was still observed. Third, although statistical models were used to adjust for potential bias, results were not adjusted for ethnicity, which might have influenced the outcomes. Fourth, the actual use of ARB/ACEI could not be ascertained from medical records, which only report whether or not the patient was prescribed these medications. Fifth, some lifestyle habits, such as alcohol use and adherence to medications/level of blood pressure control, were not available or were not collected in this study. This could potentially act to confound some of the associations observed in this study. Sixth, aiming to preserve the power of the study, the analyses considered both ACEI and ARB users combined for outcome estimates. However, when analyzed independently, use of ACEIs or ARBs were also associated with reduced mortality (OR 0.557, 95% CI 0.315 to 0.987, p = 0.045 and OR 0.388, 95% CI 0.189 to 0.797, p = 0.01; respectively).

Importantly, we addressed the limitation of lacking data regarding smoking status among medical records by conducting a sensitivity analysis. The analysis considering all patients with unknown smoking status as smokers showed that smokers had a significantly higher odds of mortality. This effect, however, disappeared after removing the patients who did not have data regarding smoking status. The observation that patients who smoke have increased mortality is supported by several studies.^26^Recently, a study by Lowe et al. (2021) showed that cumulative exposure to cigarette smoke was an independent risk factor for hospital admission and COVID-19–related mortality. Furthermore, the effect of smoking on mortality exhibited a dose-dependent relationship because patients with greater cigarette pack-years exhibited higher odds of COVID-19–related death.^27^

## Conclusion

These results suggest that hospitalized patients with COVID-19 and overweight/obesity-related hypertension who were prescribed ARB/ACEI before admission to the hospital exhibit lower length of stay and COVID-19–related mortality as compared to those not taking ARB/ACEI medications. This observation may carry important clinical implications in terms of COVID-19 treatment in this unique group of patients with overweight/obesity.

## Supplementary materials

Supplementary Tables S1 through S6 index and contain additional data as follows: S1: Predictors of mortality between patients with COVID-19 and overweight/obesity-related hypertension in ARB/ACEI and comparison groups with unknown smoking status removed. S2: Predictors of length of stay in hospital between patients with COVID-19 and overweight/obesity-related hypertension in ARB/ACEI and comparison groups with unknown smoking status removed. S3: Predictors of intensive care between patients with COVID-19 and overweight/obesity-related hypertension in ARB/ACEI and comparison groups. S4: Predictors of mechanical ventilation between patients with COVID-19 and overweight/obesity-related hypertension in ARB/ACEI and comparison groups. S5: Predictors of vasopressor use between patients with COVID-19 and overweight/obesity-related hypertension in ARB/ACEI and comparison groups. S6: Predictors of supplemental oxygen use between patients with COVID-19 and overweight/obesity-related hypertension in ARB/ACEI and comparison groups.

## * Supplemental Materials

For additional information, please click here .
